# Identifying variants that contribute to linkage for dichotomous and quantitative traits in extended pedigrees

**DOI:** 10.1186/1753-6561-5-S9-S68

**Published:** 2011-11-29

**Authors:** Wei-Min Chen, Ani Manichaikul, Stephen S Rich

**Affiliations:** 1 Center for Public Health Genomics, University of Virginia, West Complex, 6th Floor, Suite 6111, PO Box 800717, University of Virginia, Charlottesville, VA 22908, USA; 2 Department of Public Health Sciences, Division of Biostatistics and Epidemiology, University of Virginia, Charlottesville, VA 22908, USA

## Abstract

Compared to genome-wide association analysis, linkage analysis is less influenced by allelic heterogeneity. The use of linkage information in large families should provide a great opportunity to identify less frequent variants. We perform a linkage scan for both dichotomous and quantitative traits in eight extended families. For the dichotomous trait, we identified one linkage region on chromosome 4q. For quantitative traits, we identified two regions on chromosomes 4q and 6p for Q1 and one region on chromosome 6q for Q2. To identify variants that contribute to these linkage signals, we performed standard association analysis in genomic regions of interest. We also screened less frequent variants in the linkage region based on the risk ratio and phenotypic distribution among carriers. Two rare variants at *VEGFC* and one common variant on chromosome 4q conferred the greatest risk for the dichotomous trait. We identified two rare variants on chromosomes 4q (*VEGFC*) and 6p (*VEGFA*) that explain 12.4% of the total phenotypic variance of trait Q1. We also identified four variants (including one at *VNN3*) on chromosome 6q that are able to drop the linkage LOD from 3.7 to 1.0. These results suggest that the use of classical linkage and association methods in large families can provide a useful approach to identifying variants that are responsible for diseases and complex traits in families.

## Background

Common variants have been successfully identified for many diseases and complex traits through the use of genome-wide association studies (GWAS). Although many of the findings from GWAS have been replicated in different populations, current association results have yet to explain many existing linkage regions of interest. Furthermore, GWAS have limited power to identify rarer variants, in contrast to linkage analysis, which is not sensitive to allelic heterogeneity [1]. Thus the use of linkage information in family data (especially in large pedigrees) provides great opportunities to identify rarer variants.

In this paper, we present our analysis of the Genetic Analysis Workshop 17 (GAW17) family data [2] (the first set of simulated family data, without knowledge of the underlying simulating model). Our data consist of one dichotomous trait (with 30% of individuals being affected) and three quantitative traits for 697 individuals from 8 extended families. Each individual has genotypes at 24,487 single-nucleotide polymorphisms (SNPs) across 22 autosomes, and variants at more than 85% of these SNPs are either rare (minor allele frequency [MAF] < 0.01) or less frequent (0.01 < MAF < 0.05). The GAW17 family data are rich in relative pairs (579 sib pairs, 2,430 second-degree relative pairs, and thousands of other types). Each family includes four generations, with family size varying from 73 to 128. For the dichotomous trait, there are 48 affected sib pairs, 251 affected second-degree relative pairs, 202 affected third-degree relative pairs, 43 affected fourth-degree relative pairs, and 5 affected fifth-degree relative pairs. The use of more distantly related relative pairs has the potential to increase the power to identify rare, infrequent, and common variants.

Rather than breaking large pedigrees into smaller ones and then applying a standard software package (such as Merlin [3]), in this analysis we develop new implementations to perform genome-wide linkage scans in large pedigrees for both dichotomous and quantitative traits. We investigate the association of variants in the identified linkage regions.

## Methods

### Linkage and association methods for dichotomous traits

For the dichotomous trait, we use an affected relative pair nonparametric linkage (NPL) scan. The NPL method assumes that, in the genomic region in linkage with the trait, affected relative pairs are expected to share more alleles identical by descent (IBD) than average. Let *π_ij_* denote the proportion of alleles shared IBD between relatives *i* and *j* at a locus. *π_ij_* can be estimated as  based on all genotype information [3]. The expected value of *π_ij_* is twice the kinship coefficient *f_ij_* (e.g., *d*th-degree relative pairs have a kinship coefficient 1/2*^d^*^+1^ in an outbred family). For highly informative markers, the mean and variance of  are approximately 2*ϕ_ij_* and var(*π_ij_*) (e.g., 1/8 for full siblings), respectively, under the null hypothesis of no linkage. It is straightforward to consider the *Z* statistic:(1)

where (*i*, *j*) and (*k*, *l*) index all affected relative pairs. The covariance of the estimated IBD sharing can be well approximated with the technique used in the regression-based quantitative trait linkage method [4]. In the special case in which the exact IBD sharing is known (i.e., ), as in our simulated GAW17 families, it is sufficient to compute Σ*_i_*_,_*_j_*Σ*_k_*_,_*_l_* Cov(*π_ij_*, *π_kl_*), which is a function of the pedigree structure (an algorithm described by Chen and Abecasis [5]). Because it is likely that more distantly related affected relative pairs will provide more information for identification of rare variants, we consider a weight:(2)

for each affected relative pair (parent-offspring pairs are excluded for the lack of variation). Because the covariate Age is a strong risk factor for the dichotomous trait (a 10-year increase in age doubles the risk of being affected), it is crucial to take into account the effect of aging in the NPL analysis. We include only affected relative pairs with an age difference no larger than 16 years in the NPL analysis. Although this threshold is somewhat arbitrary, we subsequently applied other threshold values to ensure that our results were not too sensitive to this value. Note that our linkage results are not inflated by potential linkage disequilibrium (LD) between adjacent markers, because the IBD statistics are known in our simulated data. In practice, IBD statistics need to be estimated, and the LD needs to be properly modeled in the linkage analysis when genotype data for the parents of affected sib pairs are not complete [6].

To examine the association of SNPs with simulated phenotypes, we apply the standard transmission disequilibrium test (TDT) [7] and the more recent generalized disequilibrium test (GDT) [8] to the simulated data. The GDT is able to make use of all discordant relative pairs in extended pedigrees to compare the allele frequency differences between affected and unaffected individuals within families. Given the potential lack of power of existing association methods to detect rare variants, we developed a strategy to screen rare and infrequent variants in the linkage region. At each SNP with MAF < 0.05, we compute the odds of being affected among carriers of the variant. If the odds among carriers of the variant are much larger (or smaller) than the overall odds, we perform follow-up analyses for this SNP and consider the overall effect of the collapsed rare and infrequent variants.

### Linkage and association methods for quantitative traits

For the quantitative traits, we implement a score-based robust linkage analysis [9]. Although our test statistic is identical [10] with that in the regression-based method [4], our software implementation allows much larger pedigrees than the Merlin-regress software (the key element Cov(*π_ij_*, *π_kl_*) in the test statistic can be conveniently calculated as a function of the pedigree structure without using the SNP data). Covariates in the linear regression of the quantitative traits include Age, Smoking status, and the first principal component from a multidimensional scaling (MDS) structure analysis [11] in which the family structure is incorporated. These covariates are adjusted in the robust score test.

We examine the identified linkage region using the variance component score test as implemented in the GDT software package (through parameter fastAssoc) [12]. Although the algorithm implemented in the GDT package is identical to the one that is implemented in Merlin [3], the GDT implementation can handle much larger pedigrees because the (time-consuming) Lander-Green algorithm [13] is a required component of the Merlin package but not in GDT. To adjust for the most significant SNPs, we perform additional association scans. Finally, we fit a variance component model to estimate the unexplained heritability and the effect of each variant associated with the quantitative trait.

## Results and discussion

For the dichotomous trait, Age is the most statistically significant risk factor (*p* = 2 × 10^−16^), with a 10-year increase in age doubling the risk of being affected. Smoking is the second largest risk factor. Sex is not significantly associated with the affection status. Our NPL scan on 22 autosomes with adjustment for Age revealed one significant linkage region on chromosome 4q with maximum LOD = 3.1 at position 170.34 Mb and a one-LOD support interval between 142.8 Mb and 177.9 Mb. The linkage evidence is derived primarily from the largest family (family 7, consisting of 128 individuals). Family 7 alone provides the maximum LOD (4.2) in a region between 153.57 Mb and 177.90 Mb.

The evidence supporting linkage remains strong even with a variable age threshold. When Age is not incorporated into the NPL analysis, the maximum LOD is 2.5 in the same region. We also identified a region on chromosome 9 with suggestive evidence in support of linkage (maximum LOD = 2.9 in a region between 4.5 Mb and 7.0 Mb). However, this result is sensitive to the age threshold. When Age is not incorporated into the analysis, the evidence supporting linkage dropped to LOD = 1.7; thus we restricted our subsequent analyses to the region on chromosome 4q.

To localize the variants that contribute to the evidence supporting linkage, we first performed TDT and GDT analyses. No significant association was found (no associations have a *p* < 0.001). Rather than comparing allele frequency differences between affected and unaffected individuals, we computed the odds of being affected among carriers of rare and infrequent variants. We estimated allele frequencies based on all 202 founders, representing the general population. In the linkage region, four SNPs had odds greater than or equal to 1: C4S4373 at 167.01 Mb (odds = 9/6, MAF = 0.002), C4S4915 at 176.14 Mb (odds = 10/8, MAF = 0.005), C4S4916 at 176.14 Mb (odds = 9/6, MAF = 0.002), and C4S4935 at 177.85 Mb (odds = 16/15, MAF = 0.002). C4S4373 and C4S4916 (9 Mb apart) are in complete LD (*r*^2^ = 1, *D′* = 1) so only one of these two SNPs needed to be further studied. In addition, C4S4915 and C4S4916 (101 bp apart) are in strong LD. All C4S4916 variant carriers were also C4S4915 variant carriers, whereas three of the C4S4915 carriers were not C4S4916 carriers. All C4S4916 and C4S4935 carriers were private to family 7 and were not present in other families.

Given this strong LD, we eliminated two SNPs from the list and examined only two SNPs: C4S4916 and C4S4935. Only five individuals carry both variants. For each of the two variants, all carriers share the same copy of the rare allele (the number of alleles IBD is 1 between any pair of carriers whose relationship is second degree and above), indicating that all carriers inherited the rare variant from a common founder in family 7. When we focused only on family 7, by screening all SNPs, we also found two common variants with a large affected/unaffected ratio: C4S5105 and C4S5108. These two SNPs are 240 bp apart and in high LD; we selected C4S5108 because it provides a slightly higher significance. Together, these three variants are able to explain 36 out of 50 affected individuals in family 7. We summarize the three variants in Table 1.

**Table 1:** Variants in the chromosome 4q linkage region that are associated with the dichotomous trait in family 7

	Family 7		Carriers					
	
			C4S4916		C4S4935		C4S5108	
	
	Affected	Unaffected	Affected	Unaffected	Affected	Unaffected	Affected	Unaffected
*N*	50	78	9	6	16	15	20	18
Age (SD)	51.5 (21.0)	34.3 (12.9)	45.3 (21.3)	29.8 (9.5)	39.9 (19.8)	26.6 (9.9)	51.5 (21.9)	34.9 (12.5)
Number of smokers	17	15	2	1	4	2	8	3

The three variants explain risk in 36 out of 50 affected individuals in family 7.

We performed a logistic regression analysis with the three SNPs included in the model (Table 2). We used a robust sandwich estimator [14] to account for familial correlations. The odds ratios of the three variants were 5.35 (C4S4916, at gene *ADAM29*), 6.80 (C4S4935, *VEGFC*), and 1.77 (C4S5108). The LD block between C4S4373 and C4S4916 (9 Mb apart) together with the other two variants can explain the large interval of support in the linkage analyses.

**Table 2:** Logistic regression model for the dichotomous trait

	Position	MAF	Gene	Estimate	SE	Odds ratio	*Z*	*P*
Age				0.077	0.007	1.08	11.8	2 × 10^−16^
Smoke				0.947	0.225	2.58	4.20	2.6 × 10^−5^
C4S4916	176.135386	0.002	*ADAM29*	1.68	0.69	5.35	2.43	1.5 × 10^−2^
C4S4935	177.845572	0.002	*VEGFC*	1.92	0.46	6.80	4.14	3.5 × 10^−5^
C4S5108	185.457549	0.131	*LOC391722*	0.57	0.20	1.77	2.91	3.6 × 10^−3^

In addition to the SNP-by-SNP association analysis, the rare variants with larger effect identified through our screening procedure were collapsed within genes before the association analysis. We assumed that the collapsed genotype of an individual was a heterozygote if the individual was a carrier of any of the rare variants (a homozygote with two copies of the rare variant is rather rare). However, the GDT *p*-values were not substantially improved using the collapsed alleles.

The MDS structure analysis [11] identified population substructure among founders, and principal components for nonfounders were approximated according to their relationship relative to founders. We found that one principal component was sufficient to represent the substructure in the data. The first principal component was included in the analysis as a covariate with Age and Smoking status for the quantitative traits. A polygenic analysis [15] demonstrated that all quantitative traits were highly heritable, with *h*^2^ = 0.615 (Q1), *h*^2^ = 0.432 (Q2), and *h*^2^ = 0.697 (Q4). A bivariate analysis identified a modest genetic correlation between Q1 and Q2 (*r_G_* = 0.255), suggesting that these two traits may share genes in common. There was no evidence of a common genetic basis between Q4 and the other two quantitative traits.

The robust quantitative trait linkage analysis identified two linkage regions for Q1 and one linkage region for Q2. The first region for Q1 is on chromosome 4q, overlapping with the linkage region for the dichotomous trait. The maximum linkage support is LOD = 14.8 at 167.1 Mb, with a wide region of support (88.0–186.1 Mb with LOD > 3). The second region supporting linkage for Q1 is on chromosome 6p (LOD = 9.1, 25.6–26.4 Mb) with a large support region (LOD > 3 from 0 to 80.0 Mb). The region supporting linkage for Q2 is on chromosome 6q at position 143.6 Mb, with a maximum LOD of 3.7.

Our association scan identified rare variants on chromosomes 4p (C4S4935, at gene *VEGFC*, MAF = 0.002, *p* = 2.3 × 10^−15^) and 6p (C6S2981, at gene *VEGFA*, MAF = 0.007, *p* = 8.1 × 10^−16^). A conditional linkage analysis adjusting for these two SNPs resulted in a maximum LOD of 0 in these two regions, with no other significant associations. Table 3 shows the variance component regression model for Q1.

**Table 3:** Variance component regression model for Q1

	Chromosome	Position	MAF	Gene	Estimate	SE	*Z*	*P*
Age					0.018	0.001	18.00	1.9 × 10^−72^
Smoke					0.44	0.065	6.77	1.3 × 10^−11^
PC1					1.91	0.729	2.62	8.8 × 10^−3^
C4S4935	4	177.845572	0.002	*VEGFC*	1.64	0.207	7.92	2.3 × 10^−15^
C6S2981	6	43.854181	0.007	*VEGFA*	1.24	0.154	8.05	8.1 × 10^−16^

The estimated heritability was reduced with the two rare variants included, from *h*^2^ = 0.615 to *h*^2^ = 0.491, consistent with the two variants explaining 12.4% of the total phenotypic variance. In the largest family (family 7), the two variants explained 26.7% of the total phenotypic variance (the estimated heritability was reduced from 0.814 to 0.547). We screened the rare variants according to the phenotypic distribution of the carriers, but we did not identify any other rare variants that contributed independently to the variation beyond that from C4S4935 and C6S2981. On chromosome 6p, besides C6S2981 with a large effect on Q1, other potential rare variants include C6S752 at 25.83 Mb, C6S2245 at 31.71 Mb, and C6S2432 at 32.92 Mb; however, all C6S752 carriers (and all except one C6S2245 carrier) are also C6S2981 carriers, and all C6S2432 carriers are also C6S752 carriers. Thus a LD block exists in the region between 25.83 Mb and 43.85 Mb. This LD block could explain why the SNP with the strongest effect is at 43.85 Mb even though the linkage peak is at 26 Mb.

Q2 exhibits significant linkage on chromosome 6q (with a maximum LOD = 3.7 at 143.6 Mb). By screening the phenotypic distribution among the carriers, we identified two rare variants, one less frequent variant, and one common variant that partly explained the evidence for linkage in this region: C6S5449 at 133.1 Mb (MAF = 0.005, at gene *VNN3*), C6S6047 at 144.8 Mb (MAF = 0.012, at gene *UTRN*), C6S6659 at 155.5 Mb (MAF = 0.064, at gene *TIAM2*), and C6S6839 at 155.6 Mb (MAF = 0.002, at gene *TIAM2* private to family 4). When the four SNPs were adjusted in the linkage analysis, the LOD was reduced from 3.7 to 1.0. Although the four variants were able to explain most of the linkage in the region, they had small effects on Q2: They explained only 4.3% of total phenotypic variance, and none of the variants were significantly associated with Q2.

The linkage region and the candidate variants established in this work could be crucial for future functional analysis. The LD confounds the choice of functional SNPs. Functional analysis that follows up on this work could further determine the functional variants in the region.

## Conclusions

Our linkage and association analysis identified two rare variants (at *VEGFC*) and one common variant on chromosome 4q for the dichotomous trait, two rare variants on chromosomes 4q (at *VEGFC*) and 6p (at *VEGFA*) that explain 12.4% (or 26.7% in the largest family) of the phenotypic variance of trait Q1, and rare variants at *VNN3* on chromosome 6q that explain the linkage to trait Q2. No linkage regions were identified for trait Q4. The variant at *VEGFC* on chromosome 4q underlies both the dichotomous trait and the quantitative trait Q1. Compared to the true model that was used to simulate the GAW17 family data [2], our linkage and association findings for all four traits are confirmed.

Although linkage could be due to a single variant in a gene, as in the simulated GAW17 family data, given no prior knowledge of the genetic model, we should not rule out the possibility that linkage could be due to multiple variants in a gene. Therefore screening for rarer variants with larger effect is crucial for the association analysis. Our current screening procedure, which is based on the odds of being affected among carriers, is somewhat preliminary. Some further improvement could consider the likelihood of affection status among carriers (which gives less weight to a small number of carriers). Bioinformatic annotation (e.g., nonsynonymous SNPs only) should be incorporated as well.

The variants identified in our analysis are rare in the general population (e.g., 1 out of 404) and would be difficult to identify in a population-based study. These variants have a much higher frequency in some families, and our work shows that the use of linkage and association in large families provides a powerful way to identify variants that are responsible for diseases and complex traits.

## Competing interests

The authors declare that there are no competing interests.

## Authors’ contributions

WC designed and carried out the statistical analysis, and drafted the manuscript. AM participated in the design of the analysis and drafted the manuscript. SSR helped to draft the manuscript. All authors read and approved the final manuscript.
